# Early Upper Limb Motor Impairment in Multiple Sclerosis Across EDSS Levels: A Case–Control Study

**DOI:** 10.1002/brb3.71529

**Published:** 2026-07-31

**Authors:** Aitor Blázquez‐Fernández, Selena Marcos‐Antón, Cecilia Estrada‐Barranco, Patricia Martín‐Casas, Rosa María Ortiz‐Gutiérrez, Carmen Jiménez‐Antona, Sofía Laguarta‐Val, Roberto Cano‐de‐la‐Cuerda

**Affiliations:** ^1^ International PhD School Rey Juan Carlos University Madrid Spain; ^2^ Asociación de Leganés de Esclerosis Múltiple (ALEM) Leganés Madrid Spain; ^3^ Faculty of Health Sciences Universidad Villanueva Madrid Spain; ^4^ Department of Physical Therapy Universidad Europea de Madrid Madrid Spain; ^5^ Department of Physical Therapy Complutense University Madrid Spain; ^6^ Faculty of Health Sciences Department of Physical Therapy, Occupational Therapy, Physical Medicine and Rehabilitation Rey Juan Carlos University Madrid Spain; ^7^ Faculty of Health Sciences Motion Analysis Biomechanics Ergonomy and Motor Control Laboratory (LAMBECOM) Rey Juan Carlos University Madrid Spain

**Keywords:** assessment, Expanded Disability Status Scale (EDSS), functionality, multiple sclerosis, upper limb

## Abstract

**Background:**

Upper limb (UL) involvement is common from the early stages in people with multiple sclerosis (PwMS) and has a significant impact on daily activities, quality of life, and autonomy. However, it may be underestimated by the Expanded Disability Status Scale (EDSS).

**Aim:**

The objective of this study was to determine differences in UL function between PwMS, stratified by EDSS ≤3.5 and >3.5, and healthy controls (HCs), as well as between PwMS subgroups according to EDSS score.

**Methods:**

A case–control study was conducted following STROBE guidelines. Participants with MS were stratified into two groups on the basis of disease severity (EDSS ≤3.5 and >3.5). UL function was assessed using the hand grip strength (HGS) test, Box and Block Test (BBT), Nine‐Hole Peg Test (NHPT), and ABILHAND questionnaire. Additionally, fatigue and disease impact were evaluated using the Fatigue Severity Scale (FSS) and the Multiple Sclerosis Impact Scale 29 (MSIS‐29). HCs were assessed using the HGS test, BBT, and NHPT.

**Results:**

PwMS (*n* = 69; median age 49 years [interquartile range—IQR 13]; 66.7% female; 34 relapsing–remitting MS, 21 secondary progressive MS, and 14 primary progressive MS; median EDSS 6.0 [IQR 2.5]) showed significant impairments in all objective UL measures compared with HC (*n* = 23; median age 44.5 years [IQR 32]; 56.5% female), even at low levels of neurological disability. Differences between EDSS subgroups were observed in BBT and NHPT for the more affected UL, whereas no significant difference was found for the HGS test. For the less affected UL, only NHPT differed significantly between EDSS subgroups. No statistically significant differences were found between EDSS subgroups for ABILHAND, FSS, or MSIS‐29.

**Conclusions:**

These findings suggest that EDSS alone may not adequately capture UL dysfunction. The use of objective, sensitive, and validated assessments is essential to detect UL deficits, including subtle impairments, from early stages of MS, supporting the need for a multidimensional approach to UL disability assessment.

## Graphical abstract

Upper limb function is impaired in people with multiple sclerosis compared with healthy controls, even at low levels of neurological disability. Patients are stratified by Expanded Disability Status Scale (EDSS) score (≤3.5 and >3.5) and assessed using objective upper limb measures. Differences between EDSS subgroups are observed mainly in dexterity tests, whereas hand grip strength does not differ significantly. These findings suggest that EDSS alone may not adequately capture upper limb dysfunction even at early stages of the disease.

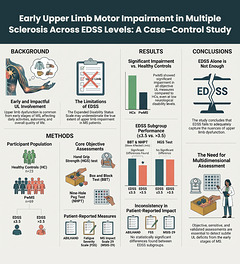

## 1 Introduction

Multiple sclerosis (MS) is a chronic, inflammatory, neurodegenerative, and autoimmune disorder of the nervous system. It is characterized by demyelinating lesions (plaques) resulting from aberrant lymphocyte activation, with relative preservation of gray matter. Although its etiology remains uncertain, it is broadly accepted that environmental factors interact with underlying genetic susceptibility (Multiple Sclerosis International Federation 2020). On the basis of its clinical course, several disease phenotypes have been delineated, including relapsing–remitting MS (RRMS), secondary progressive MS (SPMS), primary progressive (PPMS), and progressive–relapsing (PRMS), which differ in prognosis and may evolve from one phenotype to another over the course of the disease (Müller et al. 2023).

Globally, the prevalence of MS has shown a steady increase. According to the most recent *Atlas of MS* report, approximately 3,000,000 individuals are currently living with the disease worldwide. Incidence is higher among women, with a female‐to‐male ratio of 2:1, and the mean age at diagnosis is approximately 32 years (Klineova and Lublin 2018; Multiple Sclerosis International Federation 2020). The pooled incidence rate is estimated in 2.1 per 100,000 people/year. Some authors estimate that someone in the world is diagnosed with MS every 5 min (Walton et al. 2020).

MS manifests with a broad spectrum of symptoms that vary according to the extent and location of neurological involvement. Common clinical features include pain, fatigue (both physical and cognitive), and impairments in motor, sensory, urinary, and sexual functions, among others (Multiple Sclerosis International Federation 2020). Cognitive and emotional disturbances are also frequent and may significantly impact patients’ well‐being, often contributing to diminished self‐esteem (Walton et al. 2020).

People with MS (PwMS) experience substantial activity limitations that reduce functional independence, limit community participation, and decrease employability [6]. The ability to perform activities of daily living (ADLs) progressively declines as the disease advances, thereby adversely affecting quality of life (Pisa et al. 2022; Dehghani et al. 2019). The preservation of ADLs performance is compromised by a wide range of neuromuscular and motor control impairments, including altered muscle activation; reduced strength, power, and endurance; tremor; spasticity; increased fatigue; postural instability; deficits in reach and grasp; impaired coordination; and diminished manual dexterity (Cano de la Cuerda and Collado‐Vázquez 2012; Bertoni et al. 2022). As MS progresses, these deficits become increasingly prevalent and often lead to bilateral deterioration in UL function, substantially hindering everyday tasks, such as eating, dressing, and personal hygiene. This progressive decline further exacerbates the socioeconomic burden associated with the disease over time (Marcos‐Antón et al. 2022; Gil‐González et al. 2020).

The Expanded Disability Status Scale (EDSS) is the most widely accepted instrument for assessing disability in PwMS (Kurtzke 1955; Kurtzke 1983). Originally introduced in 1955 as the Disability Status Scale (DSS), it was revised in 1983 to include intermediate steps between scores 1 and 9. On the basis of a neurological examination, the EDSS evaluates several functional systems—pyramidal, cerebellar, brainstem, sensory, bowel and bladder, visual, and mental functions—using a scale from 0 to 10, where higher scores indicate greater disability (Müller et al. 2023; D'Souza et al. 2020). However, although the EDSS is a useful and widely employed tool and is considered the gold standard for determining disease severity and provides through real‐time feedback a higher consistency, it has notable limitations in assessing UL function in MS, as its scoring system is largely dependent on ambulatory capacity. Several studies have shown that more specific upper limb (UL) assessments—such as the Nine‐Hole Peg Test (NHPT), the Box and Block Test (BBT) (Solaro et al. 2020), or technology‐based evaluations—are more sensitive and clinically representative for estimating manual disability in PwMS than the EDSS alone (Goodkin et al. 1988).

Moreover, UL involvement has been shown to be common even in the early stages of the disease (Lamers et al. 2016; Marcos‐Antón et al. 2023) and, as previously noted, is closely related to performance in ADLs, quality of life, and patient autonomy. Comprehensive assessments of UL function indicate that PwMS exhibit such impairments from the early stages of the disease (Bertoni et al. 2022). However, this impairment may not be adequately captured by the EDSS score, as UL dysfunction does not necessarily follow the same progression pattern as lower limb impairment. In this context, a study (Gervasoni et al. 2025) reported that the EDSS has limitations in detecting subtle functional deficits in PwMS at early disease stages. Its sensitivity appears low for capturing multidomain alterations—motor, sensory, cognitive, and fatigue‐related—that may already be present in patients with low EDSS scores. However, the study by Gervasoni et al. (2025) focused predominantly on the lower limbs, highlighting the need to validate these findings for UL function in PwMS, as suggested by clinical observations, by comparing patients with healthy controls (HCs) while stratifying PwMS according to disease severity. Such an approach would provide valuable insights for the development of future multidomain scales designed to assess UL function from the earliest stages of the disease, potentially independent of the gait‐related disability milestones currently captured by the EDSS.

## 2 Objective

The objective of this study was to determine differences in UL function between PwMS, stratified by EDSS ≤3.5 and >3.5, and HCs, as well as between PwMS subgroups according to EDSS score. We hypothesize that all outcome measures will be impaired, even in patients with mild EDSS scores, compared with HC subjects. Furthermore, UL function is expected to be more severely affected in the subgroup with higher EDSS scores compared with those with lower EDSS scores.

## 3 Methods

### 3.1 Study Design

A case–control study was conducted to investigate UL function in PwMS compared to HC. The study adhered to the Strengthening the Reporting of Observational Studies in Epidemiology (STROBE) guidelines for case–control studies (Von Elm et al. 2008).

Ethical approval was obtained from the Local Ethics and Research Committee of the URJC (041220246322024). All participants provided written informed consent prior to their inclusion in the study, in accordance with the principles of the Declaration of Helsinki and applicable national regulations on research involving human subjects.

### 3.2 Setting and Participants

Participants with MS were recruited from different MS Patients Associations in Madrid, Spain, whereas HC were drawn from the community, ensuring comparable sociodemographic characteristics between groups.

Inclusion criteria for PwMS were as follows: A confirmed diagnosis of MS according to the revised McDonald criteria (Thompson et al. 2018); age above 18 years; an up‐to‐date EDSS score; stable disease status; and the ability to understand instructions, as evidenced by a score of 24 or higher on the Mini‐Mental State Examination (Beatty and Goodkin 1990).

Exclusion criteria for PwMS included a diagnosis of cardiovascular, respiratory, or metabolic disease, or any other condition that could interfere with study participation; the presence of comorbid neurological or musculoskeletal disorders affecting UL function; any medical condition preventing participation in the assessment protocol; occurrence of a relapse, exacerbation, or hospitalization within the 3‐month preceding the assessment; receipt of intravenous or oral steroid treatment within 6 months prior to the study; and treatment with botulinum toxin within 6 months before study initiation.

Inclusion criteria for HC were age‐ and sex‐matched to the MS group; and no history of neurological, musculoskeletal, or systemic conditions affecting UL function.

Exclusion criteria for HC included any medical condition that could interfere with participation in the assessment protocol; and cognitive impairment that would impede understanding of instructions.

### 3.3 Variables

Demographic and clinical characteristics were recorded: age, sex distribution, and MS phenotype. All clinical assessments were administered in a single session to both PwMS and HC by three trained and specialized physiotherapists. Each assessor was consistently responsible for the same set of tests across all participants, ensuring standardized and reliable evaluation. All assessments were conducted under the same environmental conditions to minimize potential administration bias and were performed for both ULs. Both sides were assessed for all participants. In PwMS, the most affected and least affected sides were determined by the treating neurologist, whereas in HC, the dominant and nondominant sides were identified by themselves. For between‐group comparisons, the lower performing hand was selected in each group. In PwMS, this corresponded to the most impaired hand, whereas in HC, it corresponded to the nondominant hand, which showed poorer functional performance in all cases, consistent with normative data on manual dexterity (Wang et al. 2015). This approach was intended to provide a conservative comparison and avoid overestimating between‐group differences.

Hand grip strength (HGS) test: HGS was measured using the Jamar hydraulic hand dynamometer, which features a grip handle and a maximum force indicator with a dual scale in pounds (0–198 lb) and kilograms (0–90 kg). The device provides highly accurate and reproducible results due to its isometric design and hydraulic system (Figueiredo et al. 2007). Each participant performed three measurements on each hand, and the average of these three readings, expressed in kilograms, was recorded as the final value, following the recommendations of Mathiowetz et al. (1984). Hand dynamometry is widely used in MS research to assess grip strength (Severijns et al. 2016; Gatti et al. 2015; Pavan et al. 2006) and is recommended by the American Society of Hand Therapists and the Brazilian Society of Hand Therapists (Villafañe et al. 2016), being recognized as a valid and reliable objective measure of hand function in PwMS (Figueiredo et al. 2007).

BBT: This test was used to assess coordination, movement speed, and unilateral gross motor skills in both ULs. The task involves transferring as many blocks as possible from one compartment of a box to the other, crossing the midline, within 1 min. The score is determined by counting the number of blocks successfully moved; if multiple blocks are transferred simultaneously, only one is counted as valid. Higher scores indicate greater gross manual dexterity (Mathiowetz et al. 1984). BBT is a quick, simple, and reliable assessment tool, and its administration and validity have been established in people with UL impairments (Desrosiers et al. 1994).

NHPT: This test assesses fine manual dexterity and involves a plastic pegboard (25.0 cm × 12.7 cm × 2.3 cm) containing nine holes spaced 2.54 cm apart, along with nine pegs (3.2 cm long and 0.64 cm wide). Participants are instructed to place the nine pegs into the board as quickly as possible, one at a time, using only one hand, and then remove them. The test was performed for each hand, starting with the non‐affected hand. The time required to complete the second trial is used for analysis (Cuesta‐Gómez et al. 2020).

ABILHAND: This questionnaire evaluates the manual abilities of adult patients. The scale assesses an individual's capacity to perform daily activities requiring the use of the ULs (Penta et al. 2001). Responses are scored as “impossible,” “difficult,” or “easy,” with higher scores indicating greater ability to carry out ADLs involving the ULs. It has been validated for PwMS and demonstrates excellent intra‐ and inter‐rater reliability, high internal consistency, and strong convergent construct validity with the Multiple Sclerosis Impact Scale‐29 (MSIS‐29) (Barrett et al. 2013).

Fatigue Severity Scale (FSS): This scale, developed by Krupp et al. (1989), comprises nine items and is designed to assess the severity of fatigue and its impact on daily activities and lifestyle, particularly in PwMS, for whom it has been validated (Valko et al. 2008). Each item is rated on a 7‐point scale, with “1” indicating “strongly disagree” and “7” indicating “strongly agree.” Total scores range from 9 to 63, with higher scores reflecting greater fatigue severity. For analysis, the total score is also converted into a percentage format.

MSIS‐29: The MSIS‐29 specifically assesses the impact of MS on the quality of life of affected individuals. It comprises 29 items across two dimensions: physical and psychological/cognitive. Each item is scored from 1 to 5, with 5 indicating a lower perceived quality of life. The maximum scores are 100 points for the physical dimension and 45 points for the psychological/cognitive dimension (Hobart et al. 2001; Téllez et al. 2005). Both dimensions are normalized to express results as percentages. Higher scores reflect a greater impact of MS on the person's quality of life. The MSIS‐29 is quick and easy to administer (5–10 min) and has been validated in Spanish. Moreover, it has demonstrated validity and reliability compared with other assessment tools in PwMS (Gray et al. 2009).

### 3.4 Group Stratification

Participants with MS were stratified according to disease severity to examine how UL function varies across different stages of the disease. Stratification was based on the EDSS, with PwMS categorized into two groups: EDSS ≤ 3.5 (3.5 = fully ambulatory but with moderate disability in one functional system and more than minimal disability in several others; or mild disability in five functional systems) and EDSS > 3.5 points (Kurtzke 1955; Kurtzke 1983). The cut‐off was used to distinguish lower and higher levels of disability within the PwMS included in the study.

This stratification enabled the analysis of UL functional performance in relation to disease progression and facilitated comparisons with HC, with each MS severity group evaluated separately against the HC. Although previous studies have analyzed cutoff points for the lower extremity in terms of minimal walking disability (Gervasoni et al. 2025; Carpinella et al. 2021; Martin et al. 2006), a cutoff of 3.5 was established as it was considered the transition point between moderate and severe disability (D'Souza et al. 2020). However, this was an exploratory cutoff, as the hypothesis of the present study is that the disability reflected by the EDSS score may not coincide with UL impairment in PwMS.

### 3.5 Statistical Analysis

Statistical analyses were performed using IBM SPSS Statistics version 29.0.2. The distribution of continuous variables was assessed using the Shapiro–Wilk test and visual inspection of histograms and *Q*–*Q* plots. Continuous variables are presented as median and interquartile range (IQR), and categorical variables as frequencies and percentages.

Objective UL outcomes were compared among three groups: HCs, PwMS with EDSS ≤ 3.5, and PwMS with EDSS > 3.5. Welch's ANOVA was used because of unequal sample sizes and heterogeneity of variances, with Games–Howell tests for post hoc comparisons. Associations between EDSS and objective UL outcomes were examined using simple linear regression models.

Patient‐reported outcomes were compared between the two EDSS subgroups using the Mann–Whitney *U* test, as these measures were only available for PwMS.

As an additional descriptive analysis (Table S1), the proportion of PwMS showing marked NHPT impairment was calculated using the MS‐specific cut‐off described by Feys et al. (2017). A completion time slower than 33.3 s, corresponding to 0.27 pegs/s, was considered indicative of marked UL dysfunction. Percentages were calculated using the number of participants with available NHPT data as the denominator and are presented as Supporting Information.

Statistical significance was set at *p* < 0.05.

## 4 Results

The study included 92 participants, comprising 69 PwMS and 23 HC. Demographic and clinical characteristics of the sample are presented in Table 1.

**TABLE 1:** Demographic and clinical characteristics of participants by group.

Variable	Total PwMS (*n* = 69)	EDSS ≤ 3.5 (*n* = 12)	EDSS > 3.5 (*n* = 57)	HC (*n* = 23)	*p* value
Age, years, median (IQR)	49 (13)	52 (19)	51 (13)	44.5 (32)	0.199
Female sex, *n* (%)	46 (66.7)	9 (75.0)	37 (64.9)	13 (56.5)	0.546
Disease duration, years, median (IQR)	14 (14)	10 (7)	16 (15)	—	—
EDSS, median (IQR)	6.0 (2.5)	2.5 (1.0)	6.0 (1.5)	—	—
MS phenotype, *n* (%)					
RRMS	34 (49.3)	9 (75.0)	25 (43.9)	—	—
SPMS	21 (30.4)	1 (8.3)	20 (35.1)	—	—
PPMS	14 (20.3)	2 (16.7)	12 (21.1)	—	—
HGS, more affected side, kg, median (IQR)	—	23.0 (14.4)	17.0 (17.2)	34.7 (13.3)	—
HGS, less affected side, kg, median (IQR)	—	26.7 (22.7)	21.7 (13.0)	37.2 (15.0)	—
BBT, more affected side, blocks/min, median (IQR)	—	51 (23)	40 (19)	75 (14)	—
BBT, less affected side, blocks/min, median (IQR)	—	52 (33)	44 (19)	75.5 (11)	—
NHPT, more affected side, s, median (IQR)	—	27.0 (14.5)	29.6 (22.4)	20.1 (5.5)	—
NHPT, less affected side, s, median (IQR)	—	26.8 (12.2)	28.0 (11.5)	18.9 (4.4)	—

*Note*: Continuous variables are presented as median (interquartile range) and categorical variables as number (percentage). Between‐group differences in age were assessed using Welch's ANOVA, and sex distribution was compared using the chi‐square test. For HGS and BBT, higher values indicate better outcomes, whereas for NHPT, lower values indicate better outcomes.

Abbreviations: BBT, Box and Block Test; EDSS, Expanded Disability Status Scale; HC, healthy controls; HGS, hand grip strength; IQR, interquartile range; MS, multiple sclerosis; NHPT, Nine‐Hole Peg Test; PPMS, primary progressive multiple sclerosis; PwMS, people with multiple sclerosis; RRMS, relapsing–remitting multiple sclerosis; SPMS, secondary progressive multiple sclerosis.

PwMS had a median age of 49 years (IQR 13); 46 participants were female (66.7%); median disease duration was 14 years (IQR 14); and median EDSS was 6.0 (IQR 2.5). Disease course was RRMS in 34 participants (49.3%), SPMS in 21 (30.4%), and PPMS in 14 (20.3%). HC had a median age of 44.5 years (IQR 32), and 13 participants were female (56.5%). No statistically significant differences were found between groups for age (*p* = 0.199) or sex distribution (*p* = 0.546).

**FIGURE 1:**
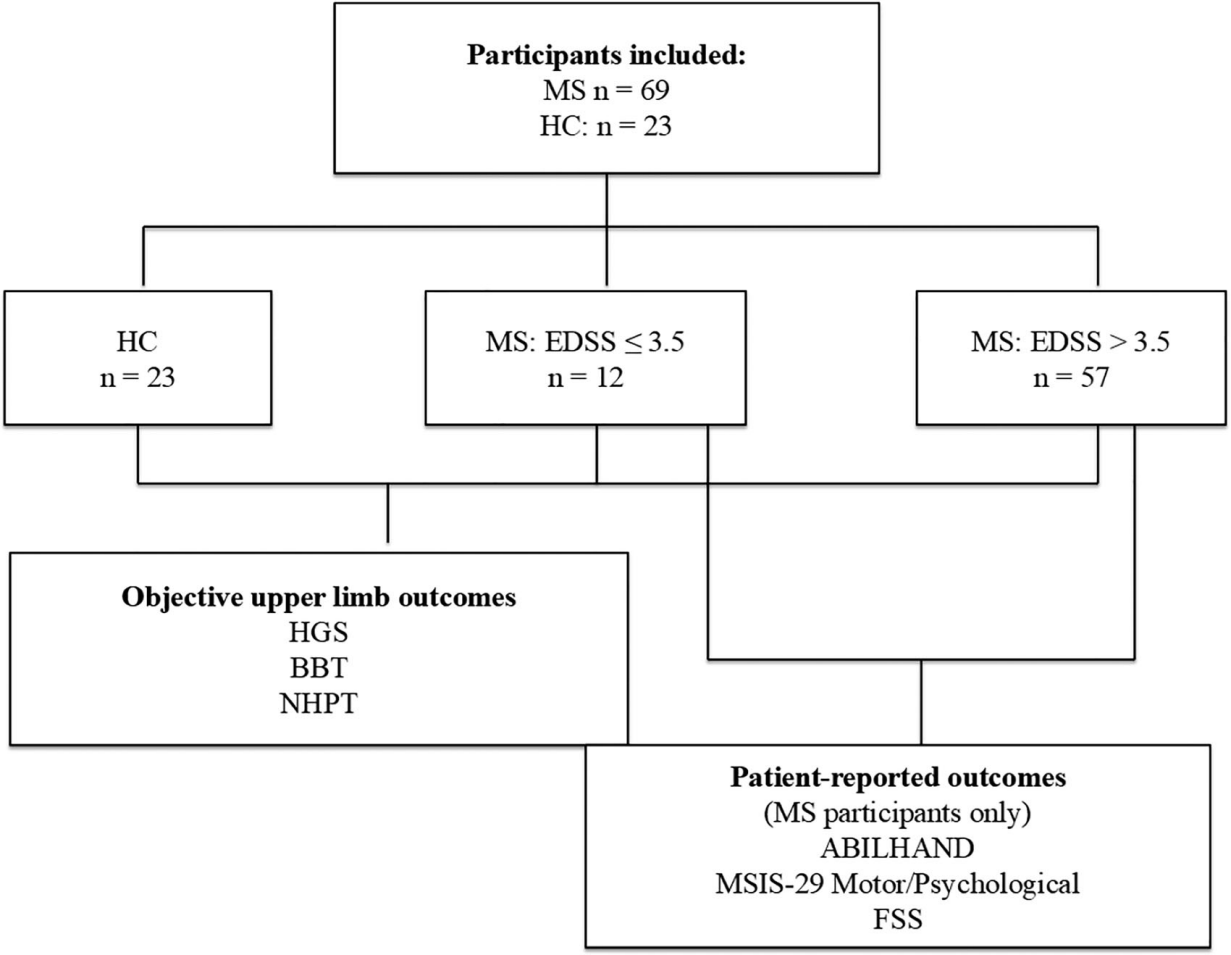
Participant flow diagram. BBT, Box and Block Test; EDSS, Expanded Disability Status Scale; FSS, Fatigue Severity Scale; HCs, healthy controls; HGS, hand grip strength; MS, multiple sclerosis; MSIS‐29, Multiple Sclerosis Impact Scale 29; NHPT, Nine‐Hole Peg Test.

PwMS were stratified according to EDSS level into EDSS ≤ 3.5 (*n* = 12) and EDSS > 3.5 (*n* = 57). The flow of participants through the study is shown in Figure 1.

UL function was compared between HC and PwMS stratified by EDSS (≤3.5 and >3.5), separately for the more affected and less affected ULs. Due to unequal sample sizes and heterogeneity of variances, Welch one‐way ANOVA was applied for all outcomes.

### 4.1 Overall Group Differences

Significant group effects were observed for all outcome measures in both the more affected and less affected UL (Table 2). For the more affected UL, significant differences were found for HGS, manual dexterity (BBT), and fine motor performance (NHPT), with large effect sizes for BBT (*η* ^2^ = 0.53) and moderate‐to‐large effects for HGS (*η* ^2^ = 0.26) and NHPT (*η* ^2^ = 0.17).

**TABLE 2:** Comparison of upper limb function according to Expanded Disability Status Scale (EDSS) group.

Upper limb	Variable	Welch F (df1, df2)	p	η2
More affected	HGS	21.33 (2, 30.57)	**<0.001**	0.26
	BBT	79.99 (2, 28.82)	**<0.001**	0.53
	NHPT	21.43 (2, 27.71)	**<0.001**	0.17
Less affected	HGS	18.12 (2, 28.03)	**<0.001**	0.25
	BBT	71.16 (2, 27.46)	**<0.001**	0.49
	NHPT	12.80 (2, 27.02)	**<0.001**	0.08

*Note*: Welch ANOVA was used to compare healthy controls and patients with multiple sclerosis stratified by EDSS (≤3.5 and >3.5) due to unequal sample sizes and heterogeneity of variances. Degrees of freedom were estimated using the Welch–Satterthwaite approximation, which results in non‐integer values and is appropriate for robust ANOVA analyses. Statistical significance was set at *p* < 0.05. Effect sizes are reported as eta squared (*η* ^2^), interpreted as small (∼0.01), moderate (∼0.06), and large (≥0.14).

Abbreviations: BBT, Box and Block Test; HGS, hand grip strength; NHPT, Nine‐Hole Peg Test.

Statistically significant results are shown in bold.

Similarly, for the less affected UL, significant group effects were observed for all outcomes (*p* < 0.001), with large effect sizes for BBT (*η* ^2^ = 0.49) and HGS (*η* ^2^ = 0.25), and a smaller but still meaningful effect for NHPT (*η* ^2^ = 0.08).

### 4.2 Post Hoc Pairwise Comparisons

Post hoc analyses using the Games–Howell procedure revealed that both EDSS subgroups (≤3.5 and >3.5) differed significantly from HC across all outcome measures and for both ULs (Table 3).

**TABLE 3:** Post hoc pairwise comparisons between groups.

Upper limb	Variable	HC vs. EDSS ≤3.5 (p)	HC vs. EDSS > 3.5 (p)	EDSS ≤ 3.5 vs. EDSS > 3.5 (p)
More affected	HGS	**0.007**	**<0.001**	0.524
	BBT	**0.001**	**<0.001**	**0.031**
	NHPT	**0.007**	**<0.001**	**0.012**
Less affected	HGS	**0.016**	**<0.001**	0.746
	BBT	**0.004**	**<0.001**	0.220
	NHPT	**0.011**	**<0.001**	**0.042**

*Note*: Post hoc pairwise comparisons were performed using the Games–Howell test following a significant Welch ANOVA. Statistical significance was set at *p* < 0.05.

Abbreviations: BBT, Box and Block Test; HC, healthy control; HGS, hand grip strength; NHPT, Nine‐Hole Peg Test.

Statistically significant results are shown in bold.

However, differences between the two EDSS subgroups were less consistent. For the more affected UL, significant differences between EDSS ≤ 3.5 and EDSS > 3.5 were observed for BBT (*p* = 0.031) and NHPT (*p* = 0.012), but not for HGS (*p* = 0.524).

For the less affected UL, a significant difference between EDSS subgroups was detected only for NHPT (*p* = 0.042), whereas no significant differences were found for HGS (*p* = 0.746) or BBT (*p* = 0.220).

A supplementary descriptive analysis based on the MS‐specific NHPT cut‐off is shown in Table S1. Overall, 31/67 PwMS (46.3%) showed marked NHPT impairment in at least one UL, including 4/11 participants (36.4%) in the EDSS ≤ 3.5 group and 27/56 (48.2%) in the EDSS > 3.5 group. These findings suggest that clinically relevant fine manual dexterity impairment may already be present in participants with lower EDSS scores.

To further examine the relationship between neurological disability and objective UL performance, simple linear regression analyses were conducted using EDSS as a continuous independent variable. Separate models were fitted for HGS, manual dexterity (BBT), and fine motor performance (NHPT) of the more affected UL (Table 4).

**TABLE 4:** Association between Expanded Disability Status Scale (EDSS) and objective upper limb performance (more affected upper limb).

Outcome	R	R2	Adjusted R2	p value
HGS	0.243	0.059	0.045	**0.046**
BBT	0.427	0.182	0.170	**<0.001**
NHPT	0.373	0.139	0.126	**0.002**

*Note*: Linear regression analyses were performed using EDSS as a continuous independent variable. Separate models were fitted for each objective measure of upper limb performance. Data correspond to the more affected upper limb. *R* indicates the Pearson correlation coefficient; *R* ^2^ represents the proportion of variance explained by EDSS; adjusted *R* ^2^ is the coefficient of determination adjusted for sample size. Statistical significance was set at *p* < 0.05.

Abbreviations: BBT, Box and Block Test; HC, healthy control; HGS, hand grip strength; NHPT, Nine‐Hole Peg Test.

Statistically significant results are shown in bold.

EDSS showed a weak but statistically significant association with HGS (*R* ^2^ = 0.059, *p* = 0.046), indicating that neurological disability explained only a small proportion of the variance in muscle strength. In contrast, moderate associations were observed for manual dexterity and fine motor performance, with EDSS explaining 18.2% of the variance in BBT performance (*p* < 0.001) and 13.9% in NHPT performance (*p* = 0.002).

Patient‐reported outcomes were compared between PwMS with EDSS ≤ 3.5 and EDSS > 3.5 using nonparametric analyses (Table 5). No statistically significant differences were observed between groups for manual ability (ABILHAND), FSS, motor impact (MSIS‐29 Motor), or psychological impact (MSIS‐29 Psychological).

**TABLE 5:** Patient‐reported outcomes according to Expanded Disability Status Scale (EDSS) subgroup (≤3.5 vs. >3.5).

Variable	EDSS ≤ 3.5 (n = 12) median (IQR)	EDSS > 3.5 (n = 57)median (IQR)	Mann–Whitney U	p value
ABILHAND	38.5 (7)	36.0 (17)	247.0	0.200
FSS	73.8 (26.6)	64.3 (35.7)	270.0	0.417
MSIS‐29 Motor	39.8 (44.6)	52.3 (41.4)	234.5	0.102
MSIS‐29 Psychological	47.8 (45.1)	45.6 (35.4)	299.0	0.552

*Note*: Data are presented as median and interquartile range (IQR). Between‐group comparisons were performed using the Mann–Whitney *U* test.

Abbreviations: FSS, Fatigue Severity Scale; MSIS‐29, Multiple Sclerosis Impact Scale 29.

## 5 Discussion

The present study was designed to comprehensively characterize UL function in pwMS across different levels of disability and to determine whether clinically meaningful impairments are already detectable in patients with mild EDSS scores when compared with HC. Our results showed that individuals with MS exhibited significant impairments across all UL assessments compared with HC, even at lower levels of neurological disability, possibly because EDSS scores primarily reflect gait‐related impairment. Moreover, differences between EDSS subgroups (≤3.5 and >3.5) were primarily evident in tasks assessing manual dexterity and fine motor performance rather than muscle strength for the more affected side and in fine manipulative tasks for the less affected side. However, it is important to note that the group with an EDSS ≤ 3.5 had a smaller sample size than the other MS subgroup, which may have influenced our findings; therefore, these results should be interpreted with caution.

By simultaneously examining multiple domains related to the UL (muscle strength, gross and fine motor coordination, fatigue, UL functional performance, and quality of life), this work addresses an important gap in current MS assessment paradigms, which remain heavily weighted toward ambulation‐based measures (Marcos‐Antón et al. 2023). Our findings provide novel evidence that UL dysfunction is present early in the disease course and spans multiple functional domains, supporting the notion that the EDSS alone may underestimate the true extent of disability linked to the UL in pwMS. These findings emphasize the need for multidimensional assessment approaches capable of detecting functionally relevant UL impairments, some of which may be subtle yet present even in the early stages of the disease, and that could directly affect daily activities and patient autonomy (van Munster et al. 2018; Dezfuli et al. 2015; Lamers et al. 2021).

Notably, few studies have specifically investigated disability in early‐stage MS, with most focusing on lower limb function and ambulation, leaving UL involvement largely underexplored. Among them, it is interesting to extract knowledge about the EDSS cut‐off points for subsequent analysis: Gervasoni et al. (2025) conducted a cross‐sectional study in 80 participants with minimal disability (EDSS < 2.5), assessing functional system scores and objective measures of lower limb strength, tactile sensitivity, balance, fatigue, and cognition. Fernández‐Vázquez et al. (2023) used 3D motion capture and statistical parametric mapping (SPM) to analyze lower limb kinematics during gait in individuals with mild disability (EDSS ≤ 3) versus HC. On the other hand, Martin et al. (2006) examined gait and balance in early MS patients with minimal disability (EDSS 0–2.5) compared with matched controls. Finally, Carpinella et al. (2021) conducted a multicenter study in early‐stage MS (EDSS ≤ 2.5) using inertial sensors during a 6‐min walk test to assess gait quality. They found that gait quality parameters (symmetry, regularity, and instability) were significantly associated with self‐reported walking limitations, and deficits were detectable even in minimally disabled patients (EDSS ≤ 1.5), whereas conventional metrics like velocity or endurance were less sensitive. It is important to note that, in all these studies, the cutoff points were defined on the basis of what is considered a hinge threshold between phases with no or minimal gait disability and scores at which gait impairment becomes moderate and/or severe. Accordingly, the choice of an EDSS cut‐off at 3.5 in the present study would be based on the hypothesis that this threshold might represent a transition from predominantly gait‐related disability toward a broader involvement of neurological functions, potentially including UL performance. Although lower EDSS scores are generally thought to reflect subtle or subclinical alterations in ambulation, scores above 3.5 could be more sensitive to the emergence of nonambulatory impairments, making this cut‐off a plausible exploratory criterion for the investigation of upper limb function. This is in line with van Munster et al. (2023). In their cohort with a median EDSS of 3.5, 87.7% of participants exhibited some level of UL impairment on the NHPT, and mild dysfunction was the most common finding across ambulation strata, indicating that deficits in UL function do not consistently align with the severity of ambulatory disability as measured by the EDSS. These observations reinforce the notion that ambulation‐centric clinical scales may underestimate broader UL functional impairment in early disease and highlight the importance of incorporating performance‐based and patient‐reported measures beyond walking ability to capture the multidimensional nature of UL disability in MS.

Our results showed that both EDSS subgroups (≤3.5 and >3.5) differed significantly from HC across all outcome measures and for both ULs. In turn, differences between the two EDSS subgroups (≤3.5 and EDSS >3.5) for the more affected UL showed significant differences for BBT and NHPT, but not for HGS. For the less affected UL, a significant difference between EDSS subgroups was detected only for NHPT, whereas no significant differences were found for HGS or BBT. These results indicate that UL dysfunction is already present in the early stages of MS and affects multiple functional domains, even when overall neurological disability is mild. The greater sensitivity of tasks assessing manual dexterity and fine motor performance to EDSS‐related differences indicates that these aspects of UL function may deteriorate earlier and more progressively than muscle strength and therefore represent more sensitive markers of disease‐related functional decline, particularly across different levels of disability and between limbs. Additionally, although EDSS was significantly associated with all objective UL measures, the proportion of variance explained was limited, particularly for muscle strength. These findings support the need to complement the EDSS with function‐specific and patient‐reported measures when assessing UL impairment in PwMS, as recommended by Cadavid et al. (2017) in the EDSS‐Plus framework. This composite endpoint, which combines the EDSS with the Timed 25‐Foot Walk and the NHPT, was shown to improve the detection of disability progression in SPMS, identifying more cases of progression than the EDSS alone and highlighting the limitations of relying exclusively on the EDSS to capture clinically relevant change. In line with this, our results are consistent with those of Solaro et al. (2020), who reported that the BBT and NHPT are more sensitive than HGS for discriminating between levels of neurological disability. In their large cohort, BBT and NHPT scores differed significantly across EDSS strata (≤3.0, 3.5–5.5, and ≥6.0), whereas HGS often failed to detect impairment, classifying 35% of patients as “normal” despite deficits in other motor domains. Our findings extend these results by showing that significant impairments across all objective measures, including strength, are already present compared with HCs in patients with mild disability (EDSS ≤ 3.5). Although Solaro et al. emphasized low correlations between these measures, our results highlight dexterity and fine motor performance (BBT and NHPT) as the key markers differentiating disability subgroups, whereas HGS remains similar between mild and moderate‐to‐severe cases. Furthermore, the limited sensitivity of HGS is reflected in our data, where the EDSS explained only 5.9% of the variance in muscle strength, compared with stronger associations for the BBT (18.2%) and NHPT (13.9%). This aligns with Solaro et al.’s observation that HGS may classify patients as unimpaired despite deficits in other domains. We also complement these objective findings with patient‐reported outcomes, which suggest a trend toward cumulative impact that may not be fully captured by clinical thresholds. Together, these studies indicate that the EDSS may underestimate true UL dysfunction, particularly in early stages, underscoring the need for multidimensional assessment approaches to capture UL deficits independent of gait‐related disability.

Our results are in line with previous authors, which point to the importance of disability related to UL function in PwMS. Johansson et al. (2007) observed that, out of 219 PwMS, 76% of patients showed UL impairments, with 50% of them experiencing moderate impairment. This appears to indicate, as observed in our results, that UL impairments are common in PwMS, gaining relevance due to their association with ADLs, which in turn impacts quality of life (Marcos‐Antón et al. 2022, 2023, 2025; Oña et al. 2022). This problem is present in more than 60% of individuals at the time of diagnosis and occurs to a greater extent from the early stages of the disease (Marcos‐Antón et al. 2023). Kamm et al. (2012) reported that after 15 years of disease progression, most PwMS experience functional impairments of the UL, leading patients to adopt compensatory behaviors or reduce task‐related functions, thereby developing compensatory strategies. Ingram et al. (2022) stated that up to 75% of PwMS report difficulties performing bimanual tasks, which constitute one of the classical targets of neurorehabilitation programs. Finally, Pisa et al. (2022) highlighted that PwMS report impairments in sensation, strength, fine manual dexterity, and gross motor function of the UL. As a result of these deficits, negative effects on employability arise, leading to adverse changes in economic status, health, and social life (Oña et al. 2022). Therefore, several authors emphasize that impairments in UL motor skills are linked to the performance of ADLs, which are also related to functional independence (Bertoni et al. 2015; Kierkegaard et al. 2012) and impact on quality of life in PwMS (Feys et al. 2017; Newsome et al. 2019; Dastan et al. 2021; Balaceanu et al. 2024). Consequently, early‐stage detection of UL impairments requires sensitive and objective assessment strategies to minimize their functional impact.

Although no statistically significant differences were observed between EDSS subgroups for patient‐reported outcomes, a clear trend toward greater impairment in perceived manual ability (ABILHAND) and motor impact (MSIS‐29 Motor) was evident with increasing EDSS scores. This pattern is consistent with the progressive nature of MS and suggests that patient‐reported measures may reflect a gradual accumulation of disability that is not fully captured by the EDSS for certain domains, particularly in relatively mildly affected populations. Altogether, these findings suggest that objective, specific, sensitive, and validated assessments can detect deficits, including subtle ones, from the early stages of the disease, reinforcing the need for multidimensional evaluation tools in the assessment of UL disability in MS.

The present study has several important clinical implications. The identification of an early, multidomain pattern of UL impairment in individuals with MS seems necessary to provide a more comprehensive approach. Our findings indicate that deficits in muscle strength, fine, and gross motor coordination are present, compared to HC, even in patients with mild EDSS scores; in this early stage, EDSS assessment—primarily focused on ambulation disability (0–10 scale)—may fail to capture these subtle yet functionally meaningful alterations. By comparing patients between EDSS subgroups (≤3.5 and EDSS > 3.5) (van Munster et al. 2023), differences were identified in manual dexterity and fine motor performance for the more affected side but not in muscle strength; on the other hand, for the less affected side, differences were only identified in fine motor performance. Our results allow us to delineate the profile of UL dysfunction across different EDSS thresholds, suggesting in our sample a possible pattern of progression of UL impairment as captured by the assessment tools employed. These findings also suggest that the EDSS cut‐off point of 3.5, traditionally used to identify the onset of moderate gait impairment, may not be optimal for capturing the pattern and timing of UL involvement, as UL dysfunction may emerge or progress earlier and independently of ambulatory deterioration. Our results underscore the potential value of integrating multidimensional UL evaluation into routine clinical practice to guide early therapeutic interventions, preserve independence in ADLs, and inform individualized rehabilitation strategies before conventional disability scores reflect substantial functional loss.

Several limitations of the present study should be acknowledged. First, the overall sample size was relatively small, with a limited number of participants representing the early stages of the disease (EDSS ≤ 3.5). This imbalance was primarily attributable to the recruitment setting, as the study sample was drawn from a patient association, where individuals with lower levels of disability are less likely to attend, whereas those with higher levels of disability are more commonly represented. Future studies should aim to include larger samples of patients with lower levels of disability, who are often underrepresented in patient associations, to ensure a more balanced and generalizable analysis across the disability spectrum. Consequently, our findings cannot be generalized to the broader population of individuals with MS. Second, the cross‐sectional design of the study precludes conclusions regarding the real temporal progression of UL impairments. Third, data regarding participants’ pharmacological treatments and/or ongoing rehabilitative interventions were not collected, which may have influenced the observed outcomes. Fourth, analyzing all the domains that comprise UL function can be difficult in a clinical setting, due to both the cost of assessment tools and the time constraints faced by the therapists. These limitations should be considered when interpreting the results and highlight the need for larger, longitudinal studies incorporating comprehensive treatment data.

## 6 Conclusions

Our results showed that PwMS exhibited significant impairments across all UL assessments compared with HC, even at lower levels of neurological disability (possibly because EDSS scores primarily reflect gait‐related impairment). Moreover, differences between EDSS subgroups (≤3.5 and >3.5) were primarily evident in tasks assessing manual dexterity and fine motor performance rather than muscle strength for the more affected side and only in fine motor performance for the less affected side. No statistically significant differences were observed between EDSS subgroups for patient‐reported outcomes, but with a clear trend toward greater impairment as increasing EDSS scores. These findings also suggest that the EDSS cut‐off point of 3.5, traditionally used to identify the onset of moderate gait impairment, may not be optimal for capturing the pattern and timing of UL involvement, as UL dysfunction may emerge or progress independently of ambulatory deterioration. Finally, objective, specific, sensitive, and validated assessments must be used to detect UL deficits, including subtle ones, from the early stages of the disease, reinforcing the need for multidimensional evaluation tools in the assessment of UL disability in PwMS.

## Author Contributions

**Aitor Blázquez‐Fernández**: conceptualization, investigation, methodology, writing – original draft. **Patricia Martín‐Casas**: formal analysis, data curation, writing – original draft. **Cecilia Estrada‐Barranco**: formal analysis, data curation, writing – original draft. **Rosa María Ortiz‐Gutiérrez**: formal analysis, data curation, writing – original draft. **Selena Marcos‐Antón**: conceptualization, investigation, writing – original draft, methodology. **Sofía Laguarta‐Val**: formal analysis, data curation, writing – original draft. **Carmen Jiménez‐Antona**: formal analysis, data curation, writing – original draft. **Roberto Cano‐de‐la‐Cuerda**: conceptualization, investigation, writing – original draft, methodology, writing – review and editing, formal analysis, data curation, supervision.

## Funding

The project has been funded through a competitive call, specifically the 6th Research Grant Call awarded by the Illustrious Professional College of Physiotherapists of the Community of Madrid (Spain). 1‐10‐2025.

## Conflicts of Interest

The authors declare no conflicts of interest.

## Supporting information




**Supplementary Table**: brb371529‐sup‐0001‐TableS1.docx

## Data Availability

The data presented in this study are available upon request from the corresponding author.

## References

[brb371529-bib-0001] Balaceanu, A. , I. Puertas , M. Alonso de Leciñana , et al. 2024. “Automatic Evaluation of the Nine‐Hole Peg Test in Multiple Sclerosis Patients Using Machine Learning Models.” Biomedical Signal Processing and Control 92: 106128.

[brb371529-bib-0002] Barrett, L. E. , S. J. Cano , J. P. Zajicek , and J. C. Hobart . 2013. “Can the ABILHAND Handle Manual Ability in MS?” Multiple Sclerosis Journal 19, no. 6: 806–815. 23095289 10.1177/1352458512462919

[brb371529-bib-0003] Beatty, W. W. , and D. E. Goodkin . 1990. “Screening for Cognitive Impairment in Multiple Sclerosis: An Evaluation of the Mini‐Mental State Examination.” Archives of Neurology 47, no. 3: 297–301. 2310313 10.1001/archneur.1990.00530030069018

[brb371529-bib-0004] Bertoni, R. , D. Cattaneo , C. Grosso , F. Baglio , and J. Jonsdottir . 2022. “Distribution and Relation of Two Arm Function Tests, Box and Blocks Test and Nine Hole Peg Test, Across Disease Severity Levels and Types of Multiple Sclerosis.” Multiple Sclerosis and Related Disorders 59: 103683. 35168094 10.1016/j.msard.2022.103683

[brb371529-bib-0005] Bertoni, R. , I. Lamers , C. C. Chen , P. Feys , and D. Cattaneo . 2015. “Unilateral and Bilateral Upper Limb Dysfunction at Body Functions, Activity and Participation Levels in People With Multiple Sclerosis.” Multiple Sclerosis Journal 21, no. 12: 1566–1574. 25662346 10.1177/1352458514567553

[brb371529-bib-0006] Cadavid, D. , J. A. Cohen , M. S. Freedman , et al. 2017. “The EDSS‐Plus, an Improved Endpoint for Disability Progression in Secondary Progressive Multiple Sclerosis.” Multiple Sclerosis Journal 23, no. 1: 94–105. 27003945 10.1177/1352458516638941

[brb371529-bib-0007] Cano de la Cuerda, R. , and S. Collado‐Vázquez . 2012. Neurorrehabilitación: Métodos Específicos de Valoración y Tratamiento. Médica Panamericana.

[brb371529-bib-0008] Carpinella, I. , E. Gervasoni , D. Anastasi , et al. 2021. “Instrumentally Assessed Gait Quality is More Relevant Than Gait Endurance and Velocity to Explain Patient‐Reported Walking Ability in Early‐Stage Multiple Sclerosis.” European Journal of Neurology 28: 2259–2268. 33864413 10.1111/ene.14866

[brb371529-bib-0009] Cuesta‐Gómez, A. , P. Sánchez‐Herrera‐Baeza , E. D. Oña‐Simbaña , et al. 2020. “Effects of Virtual Reality Associated With Serious Games for Upper Limb Rehabilitation Inpatients With Multiple Sclerosis: Randomized Controlled Trial.” Journal of NeuroEngineering and Rehabilitation 17, no. 1: 90. 32660604 10.1186/s12984-020-00718-x PMC7359450

[brb371529-bib-0010] Dastan, S. , N. A. Yapici , and A. T. Ozdogar . 2021. “Investigating the Relationship Between Balance and Upper Extremity Function in Multiple Sclerosis.” Journal of Medical Science Research 9, no. 2: 79–83.

[brb371529-bib-0011] Dehghani, A. , M. Khoramkish , and S. Shahsavari Isfahani . 2019. “Challenges in the Daily Living Activities of Patients With Multiple Sclerosis: A Qualitative Content Analysis.” International Journal of Community Based Nursing and Midwifery 7, no. 3: 201–210. 31341919 10.30476/IJCBNM.2019.44995 PMC6614347

[brb371529-bib-0012] Desrosiers, J. , G. Bravo , R. Hébert , É. Dutil , and L. Mercier . 1994. “Validation of the Box and Block Test as a Measure of Dexterity of Elderly People: Reliability, Validity, and Norms Studies.” Archives of Physical Medicine and Rehabilitation 75: 751–755. 8024419

[brb371529-bib-0013] Dezfuli, M. G. , M. Akbarfahimi , S. M. Nabavi , A. Hassani Mehraban , and E. Jafarzadehpur . 2015. “Can Hand Dexterity Predict the Disability Status of Patients With Multiple Sclerosis?” Medical Journal of the Islamic Republic of Iran 29: 255. 26793646 PMC4715381

[brb371529-bib-0014] D'Souza, M. , A. Heikkilä , J. Lorscheider , et al. 2020. “Electronic Neurostatus‐EDSS Increases the Quality of Expanded Disability Status Scale Assessments: Experience From Two Phase 3 Clinical Trials.” Multiple Sclerosis Journal 26, no. 8: 993–996. 31060429 10.1177/1352458519845108

[brb371529-bib-0015] Fernández‐Vázquez, D. , G. Calvo‐Malón , F. Molina‐Rueda , et al. 2023. “Kinematic Gait Analysis in People With Mild‐Disability Multiple Sclerosis Using Statistical Parametric Mapping: A Cross‐Sectional Study.” Sensors 23, no. 18: 7671. 37765727 10.3390/s23187671 PMC10535645

[brb371529-bib-0016] Feys, P. , I. Lamers , G. Francis , et al. 2017. “The Nine‐Hole Peg Test as a Manual Dexterity Performance Measure for Multiple Sclerosis.” Multiple Sclerosis Journal 23, no. 5: 711–720. 28206826 10.1177/1352458517690824 PMC5405844

[brb371529-bib-0017] Figueiredo, I. M. , R. F. Sampaio , M. C. Mancini , F. C. M. Silva , and M. A. P. Souza . 2007. “Test of Grip Strength Using the Jamar Dynamometer.” Acta Fisiátrica 14, no. 2: 104–110.

[brb371529-bib-0018] Gatti, R. , A. Tettamanti , S. Lambiase , P. Rossi , and M. Comola . 2015. “Improving Hand Functional Use in Subjects With Multiple Sclerosis Using a Musical Keyboard: A Randomized Controlled Trial.” Physiotherapy Research International 20, no. 2: 100–107. 25045035 10.1002/pri.1600

[brb371529-bib-0019] Gervasoni, E. , A. Torchio , R. Bertoni , et al. 2025. “Beyond EDSS: Multidomain Impairments Are Detectable and Associated With Walking Disorders in Low‐Disabled People With Multiple Sclerosis.” Journal of Neurology 272: 404. 40382461 10.1007/s00415-025-13128-7

[brb371529-bib-0020] Gil‐González, I. , A. Martín‐Rodríguez , R. Conrad , and M. Á. Pérez‐San‐Gregorio . 2020. “Quality of Life in Adults With Multiple Sclerosis: A Systematic Review.” BMJ Open 10, no. 11: e041249. 10.1136/bmjopen-2020-041249. PMC7705559 33257490

[brb371529-bib-0021] Goodkin, D. E. , D. Hertsgaard , and J. Seminary . 1988. “Upper Extremity Function in Multiple Sclerosis: Improving Assessment Sensitivity With Box‐and‐Block and Nine‐Hole Peg Tests.” Archives of Physical Medicine and Rehabilitation 69, no. 10: 850–854. 3178453

[brb371529-bib-0022] Gray, O. , G. McDonnell , and S. Hawkins . 2009. “Tried and Tested: The Psychometric Properties of the Multiple Sclerosis Impact Scale (MSIS‐29) in a Population Based Study.” Multiple Sclerosis Journal 15, no. 1: 75–80. 18829636 10.1177/1352458508096872

[brb371529-bib-0023] Hobart, J. , D. Lamping , R. Fitzpatrick , A. Riazi , and A. Thompson . 2001. “The Multiple Sclerosis Impact Scale (MSIS‐29): A New Patient‐Based Outcome Measure.” Brain 124, no. 5: 962–973. 11335698 10.1093/brain/124.5.962

[brb371529-bib-0024] Ingram, L. A. , A. A. Butler , M. A. Brodie , P. Hoang , S. C. Gandevia , and S. R. Lord . 2022. “Quantifying Upper‐Limb Motor Impairment in People With Multiple Sclerosis: A Physiological Profiling Approach.” Annals of Physical and Rehabilitation Medicine 65, no. 5: 101625. 34958919 10.1016/j.rehab.2021.101625

[brb371529-bib-0025] Johansson, S. , C. Ytterberg , I. M. Claesson , et al. 2007. “High Concurrent Presence of Disability in Multiple Sclerosis. Associations With Perceived Health.” Journal of Neurology 254, no. 6: 767–773. 17401746 10.1007/s00415-006-0431-5

[brb371529-bib-0026] Kamm, C. P. , M. R. Heldner , T. Vanbellingen , H. P. Mattle , R. Müri , and S. Bohlhalter . 2012. “Limb Apraxia in Multiple Sclerosis: Prevalence and Impact on Manual Dexterity and Activities of Daily Living.” Archives of Physical Medicine and Rehabilitation 93, no. 6: 1081–1085. 22464095 10.1016/j.apmr.2012.01.008

[brb371529-bib-0027] Kierkegaard, M. , U. Einarsson , K. Gottberg , L. von Koch , and L. W. Holmqvist . 2012. “The Relationship Between Walking, Manual Dexterity, Cognition and Activity/Participation in Persons With Multiple Sclerosis.” Multiple Sclerosis Journal 18, no. 5: 639–646. 21982871 10.1177/1352458511426736

[brb371529-bib-0028] Klineova, S. , and F. D. Lublin . 2018. “Clinical Course of Multiple Sclerosis.” Cold Spring Harbor Perspectives in Medicine 8, no. 9: a028928. 29358317 10.1101/cshperspect.a028928 PMC6120692

[brb371529-bib-0029] Krupp, L. B. , N. G. LaRocca , J. Muir‐Nash , and A. D. Steinberg . 1989. “The Fatigue Severity Scale: Application to Patients With Multiple Sclerosis and Systemic Lupus Erythematosus.” Archives of Neurology 46, no. 10: 1121–1123. 2803071 10.1001/archneur.1989.00520460115022

[brb371529-bib-0030] Kurtzke, J. F. 1955. “A New Scale for Evaluating Disability in Multiple Sclerosis.” Neurology 5, no. 8: 580–583. 13244774 10.1212/wnl.5.8.580

[brb371529-bib-0031] Kurtzke, J. F. 1983. “Rating Neurologic Impairment in Multiple Sclerosis: An Expanded Disability Status Scale (EDSS).” Neurology 33, no. 11: 1444–1452. 10.1212/wnl.33.11.1444. 6685237

[brb371529-bib-0032] Lamers, I. , L. Kerkhofs , J. Raats , et al. 2021. “Upper Limb Dexterity in Patients With Multiple Sclerosis: Relationship With Activities of Daily Living and Quality of Life.” International Journal of MS Care 23, no. 2: 79–87.33880084

[brb371529-bib-0033] Lamers, I. , A. Maris , D. Severijns , et al. 2016. “Upper Limb Rehabilitation in People With Multiple Sclerosis: A Systematic Review.” Neurorehabilitation and Neural Repair 30, no. 8: 773–793. 26747125 10.1177/1545968315624785

[brb371529-bib-0034] Marcos‐Antón, S. , R. Cano‐de‐la‐Cuerda , I. Aranda‐Reneo , A. Jardón‐Huete , E. D. Oña‐Simbaña , and J. Oliva‐Moreno . 2025. “Cost‐Effectiveness Analysis of the MYO Armband Device in Combination With Specifically Designed Video Games for Upper Limb Rehabilitation in People With Multiple Sclerosis.” Journal of NeuroEngineering and Rehabilitation 23: 52. 10.1186/s12984-025-01801-x. 41299524 PMC12866502

[brb371529-bib-0035] Marcos‐Antón, S. , M. D. Gor‐García‐Fogeda , and R. Cano‐de‐la‐Cuerda . 2022. “An sEMG‐Controlled Forearm Bracelet for Assessing and Training Manual Dexterity in Rehabilitation: A Systematic Review.” Journal of Clinical Medicine 11, no. 11: 3119. 35683503 10.3390/jcm11113119 PMC9181798

[brb371529-bib-0036] Marcos‐Antón, S. , A. Jardón‐Huete , E. D. Oña‐Simbaña , A. Blázquez‐Fernández , L. Martínez‐Rolando , and R. Cano‐de‐la‐Cuerda . 2023. “sEMG‐Controlled Forearm Bracelet and Serious Game‐Based Rehabilitation for Training Manual Dexterity in People With Multiple Sclerosis: A Randomised Controlled Trial.” Journal of NeuroEngineering and Rehabilitation 20, no. 1: 110. 37598176 10.1186/s12984-023-01233-5 PMC10440030

[brb371529-bib-0037] Martin, C. L. , B. A. Phillips , T. J. Kilpatrick , et al. 2006. “Gait and Balance Impairment in Early Multiple Sclerosis in the Absence of Clinical Disability.” Multiple Sclerosis Journal 12, no. 5: 620–628. 17086909 10.1177/1352458506070658

[brb371529-bib-0038] Mathiowetz, V. , G. Volland , N. Kashman , and K. Weber . 1985. “Adult Norms for the Box and Block Test of Manual Dexterity.” American Journal of Occupational Therapy 39: 386–391. 10.5014/ajot.39.6.386 3160243

[brb371529-bib-0039] Mathiowetz, V. , K. Weber , G. Volland , and N. Kashman . 1984. “Reliability and Validity of Grip and Pinch Strength Evaluations.” Journal of Hand Surgery 9, no. 2: 222–226. 6715829 10.1016/s0363-5023(84)80146-x

[brb371529-bib-0040] Müller, J. , A. Cagol , J. Lorscheider , et al. 2023. “Harmonizing Definitions for Progression Independent of Relapse Activity in Multiple Sclerosis: A Systematic Review.” JAMA Neurology 80, no. 11: 1232–1245. 37782515 10.1001/jamaneurol.2023.3331

[brb371529-bib-0041] Multiple Sclerosis International Federation . 2020. Atlas of MS. 3rd ed. MSIF.

[brb371529-bib-0042] Newsome, S. D. , G. von Geldern , H. Shou , et al. 2019. “Longitudinal Assessment of Hand Function in Individuals With Multiple Sclerosis.” Multiple Sclerosis and Related Disorders 32: 107–113. 31085489 10.1016/j.msard.2019.04.035 PMC6995030

[brb371529-bib-0043] Oña, E. D. , S. Marcos‐Antón , D. S. Copaci , J. Arias , R. Cano‐de‐la‐Cuerda , and A. Jardón . 2022. “Effects of EMG‐Controlled Video Games on the Upper Limb Functionality in Patients With Multiple Sclerosis: A Feasibility Study and Development Description.” Computational Intelligence and Neuroscience 2022: 3735979. 35449748 10.1155/2022/3735979 PMC9017529

[brb371529-bib-0044] Pavan, K. , K. Schmidt , T. de Ambrosio Ariça , M. F. Mendes , C. P. Tilbery , and S. Lianza . 2006. “Fatigability Evaluation on Multiple Sclerosis Patients by Using a Hand Held Dynamometer.” Arquivos de Neuro‐Psiquiatria 64, no. 2A: 283–286. 16791370 10.1590/s0004-282x2006000200020

[brb371529-bib-0045] Penta, M. , L. Tesio , C. Arnould , A. Zancan , and J. L. Thonnard . 2001. “The ABILHAND Questionnaire as a Measure of Manual Ability in Chronic Stroke Patients: Rasch‐Based Validation and Relationship to Upper Limb Impairment.” Stroke; A Journal of Cerebral Circulation 32, no. 7: 1627–1634. 10.1161/01.str.32.7.1627 11441211

[brb371529-bib-0046] Pisa, M. , J. A. Ruiz , G. C. DeLuca , et al. 2022. “Quantification of Upper Limb Dysfunction in the Activities of the Daily Living in Persons With Multiple Sclerosis.” Multiple Sclerosis and Related Disorders 63: 103917. 35671673 10.1016/j.msard.2022.103917

[brb371529-bib-0047] Severijns, D. , M. Lemmens , R. Thoelen , and P. Feys . 2016. “Motor Fatigability After Low‐Intensity Hand Grip Exercises in Persons With Multiple Sclerosis.” Multiple Sclerosis and Related Disorders 10: 7–13. 27919502 10.1016/j.msard.2016.08.007

[brb371529-bib-0048] Solaro, C. , R. Di Giovanni , E. Grange , et al. 2020. “Box and Block Test, Hand Grip Strength and Nine‐Hole Peg Test: Correlations Between Three Upper Limb Objective Measures in Multiple Sclerosis.” European Journal of Neurology 27, no. 12: 2523–2530. 32619066 10.1111/ene.14427

[brb371529-bib-0049] Téllez, N. , J. Río , M. Tintoré , C. Nos , I. Galán , and X. Montalban . 2005. “Does the Modified Fatigue Impact Scale Offer a More Comprehensive Assessment of Fatigue in MS?” Multiple Sclerosis Journal 11, no. 2: 198–202. 15794395 10.1191/1352458505ms1148oa

[brb371529-bib-0050] Thompson, A. J. , B. L. Banwell , F. Barkhof , et al. 2018. “Diagnosis of Multiple Sclerosis: 2017 Revisions of the McDonald Criteria.” Lancet Neurology 17, no. 2: 162–173. 29275977 10.1016/S1474-4422(17)30470-2

[brb371529-bib-0051] Valko, P. O. , C. L. Bassetti , K. E. Bloch , U. Held , and C. R. Baumann . 2008. “Validation of the Fatigue Severity Scale in a Swiss Cohort.” Sleep 31, no. 11: 1601–1607. 19014080 10.1093/sleep/31.11.1601 PMC2579971

[brb371529-bib-0052] van Munster, C. E. P. , B. Jessica , S. Steinheimer , et al. 2023. “Assessment of Multiple Aspects of Upper Extremity Function Independent From Ambulation in Patients With Multiple Sclerosis.” International Journal of MS Care 25, no. 5: 226–232. 37720262 10.7224/1537-2073.2021-069 PMC10503816

[brb371529-bib-0053] van Munster, C. E. P. , B. M. J. Uitdehaag , F. A. H. van der Linden , et al. 2018. “Tasks of Activities of Daily Living Are More Valuable Than Classical Neurological Tests in Assessing Upper Extremity Function and Mobility in Multiple Sclerosis.” Multiple Sclerosis and Related Disorders 25: 124–130.

[brb371529-bib-0054] Villafañe, J. H. , K. Valdes , R. Buraschi , M. Martinelli , L. Bissolotti , and S. Negrini . 2016. “Reliability of the Handgrip Strength Test in Elderly Subjects With Parkinson Disease.” Hand 11, no. 1: 54–58. 27418890 10.1177/1558944715614852 PMC4920506

[brb371529-bib-0055] Von Elm, E. , D. G. Altman , M. Egger , S. J. Pocock , P. C. Gøtzsche , and J. P. Vandenbroucke . 2008. “The Strengthening the Reporting of Observational Studies in Epidemiology (STROBE) Statement: Guidelines for Reporting Observational Studies.” Journal of Clinical Epidemiology 61, no. 4: 344–349. 18313558 10.1016/j.jclinepi.2007.11.008

[brb371529-bib-0056] Walton, C. , R. King , L. Rechtman , et al. 2020. “Rising Prevalence of Multiple Sclerosis Worldwide: Insights From the Atlas of MS, Third Edition.” Multiple Sclerosis Journal 26, no. 14: 1816–1821. 33174475 10.1177/1352458520970841 PMC7720355

[brb371529-bib-0057] Wang, Y. C. , R. W. Bohannon , J. Kapellusch , A. Garg , and R. C. Gershon . 2015. “Dexterity as Measured With the 9‐Hole Peg Test (9‐HPT) Across the Age Span.” Journal of Hand Therapy: Official Journal of the American Society of Hand Therapists 28, no. 1: 53–60. 25449717 10.1016/j.jht.2014.09.002

