# Supplementary material for: Early Upper Limb Motor Impairment in Multiple Sclerosis Across EDSS Levels: A Case–Control Study
Source: Brain Behav. 2026 Jul 31;16(8):e71529. doi: 10.1002/brb3.71529 (PMC13425605; doi:10.1002/brb3.71529)
Supplement: Supplementary file 1 — Supplementary Table: brb371529‐sup‐0001‐TableS1.docx [file BRB3-16-e71529-s001.docx]

***Supplementary Table 1.*** Percentage of PwMS showing marked NHPT impairment according to the MS-specific cut-off.

| **Variable** | **EDSS ≤3.5 n/N (%)** | **EDSS >3.5 n/N (%)** | **Total PwMS n/N (%)** |
| --- | --- | --- | --- |
| NHPT, more affected upper limb | 3/11 (27.3%) | 20/54 (37.0%) | 23/65 (35.4%) |
| NHPT, less affected upper limb | 1/11 (9.1%) | 20/56 (35.7%) | 21/67 (31.3%) |
| NHPT, at least one upper limb | 4/11 (36.4%) | 27/56 (48.2%) | 31/67 (46.3%) |

Marked NHPT impairment was defined as a completion time slower than 33.3 seconds, corresponding to the published 0.27 pegs/s cut-off for marked upper limb dysfunction in MS described by Feys et al. Percentages were calculated using participants with available NHPT data as the denominator. EDSS: Expanded Disability Status Scale; MS: multiple sclerosis; NHPT: Nine-Hole Peg Test; PwMS: people with multiple sclerosis.
